# Seagrass habitat suitability model for Redang Marine Park using multibeam echosounder data: Testing different spatial resolutions and analysis window sizes

**DOI:** 10.1371/journal.pone.0257761

**Published:** 2021-09-23

**Authors:** Muhammad Abdul Hakim Muhamad, Rozaimi Che Hasan, Najhan Md Said, Jillian Lean-Sim Ooi

**Affiliations:** 1 Razak Faculty of Technology and Informatics, Universiti Teknologi Malaysia, Kuala Lumpur, Malaysia; 2 National Hydrographic Centre, Pulai Indah, Selangor, Malaysia; 3 Department of Geography, Faculty of Arts and Social Sciences, Universiti Malaya, Kuala Lumpur, Malaysia; University of Florida, UNITED STATES

## Abstract

Integrating Multibeam Echosounder (MBES) data (bathymetry and backscatter) and underwater video technology allows scientists to study marine habitats. However, use of such data in modeling suitable seagrass habitats in Malaysian coastal waters is still limited. This study tested multiple spatial resolutions (1 and 50 m) and analysis window sizes (3 × 3, 9 × 9, and 21 × 21 cells) probably suitable for seagrass-habitat relationships in Redang Marine Park, Terengganu, Malaysia. A maximum entropy algorithm was applied, using 12 bathymetric and backscatter predictors to develop a total of 6 seagrass habitat suitability models. The results indicated that both fine and coarse spatial resolution datasets could produce models with high accuracy (>90%). However, the models derived from the coarser resolution dataset displayed inconsistent habitat suitability maps for different analysis window sizes. In contrast, habitat models derived from the fine resolution dataset exhibited similar habitat distribution patterns for three different analysis window sizes. Bathymetry was found to be the most influential predictor in all the models. The backscatter predictors, such as angular range analysis inversion parameters (characterization and grain size), gray-level co-occurrence texture predictors, and backscatter intensity levels, were more important for coarse resolution models. Areas of highest habitat suitability for seagrass were predicted to be in shallower (<20 m) waters and scattered between fringing reefs (east to south). Some fragmented, highly suitable habitats were also identified in the shallower (<20 m) areas in the northwest of the prediction models and scattered between fringing reefs. This study highlighted the importance of investigating the suitable spatial resolution and analysis window size of predictors from MBES for modeling suitable seagrass habitats. The findings provide important insight on the use of remote acoustic sonar data to study and map seagrass distribution in Malaysia coastal water.

## Introduction

Seagrass ecosystems provide many critical ecological functions that support the well-being and livelihoods of local communities [1, 2]. Seagrass ecosystems provide food for many marine species [3, 4] and serve as nursery grounds for fishes [5, 6]. They are also known for their capacity to produce and export organic carbon, regulate carbon dioxide through photosynthesis, absorb and recycle nutrients, stabilize sediment, reduce coastal erosion [7–9], and reduce pathogens and disease prevalence in neighboring coral reefs [9]. Seagrass occurs in tropical and temperate waters and is generally found in coastal subtidal and intertidal areas [10]. In Malaysia, seagrass is distributed into four primary regions on the west and east coast of Peninsular Malaysia and Sabah and Sarawak in East Malaysia [11–15]. The coast of Peninsular Malaysia is relatively broad, and ecological conditions vary on the east and west coasts. Seagrasses are usually found in calm lagoons on the west coast, sheltered from the open sea behind the tidal sand ridges. On the east coast, they are found on offshore islands (e.g., Sibu Island, Tinggi Island, and Redang Island) with fringing corals and colonize the outer coastal area between the coral and semi-open seas. On the west coast, seagrass can be found in open sea coastal waters. In Sabah, patches of seagrass beds are found extensively along the west coast (e.g., Bak-Bak, Tanjung Mengayau, Sepangar Bay, and Gaya Island), south-eastern coast, and offshore islands (e.g., Sipadan, Maganting, Tabawan, and Bohey Dulang). In Sarawak, seagrasses are normally scattered near river estuaries (e.g., Bintulu River, Punang–Sri Lawas River estuary). Furthermore, seagrass also inhabits deep subtidal areas and intertidal sandy, rocky shores on Kuala Similajau, Bintulu, Sarawak.

Seagrass ecosystems are susceptible to both natural and anthropogenic threats [16, 17]. Malaysian seagrass ecosystems continually face serious threats from natural causes (e.g., erosion, flooding, surface water temperature, and turbidity) and human activities (e.g., dynamite fishing, sand mining, dredging, settlement, and construction) that cause significant degradation and possible habitat loss [17, 18]. The Sungai Pulai estuary in Johor is a threat that has arisen because of the development of coastal zones and islands. The development involves sand mining, filling, and land reclamation, which have immediately and substantially affected the marine environment and its resources. Land reclamation has led to heavy loads of suspended sediments, which often deposit a layer of silt that are several centimeters deep over the seagrass and benthic communities [18]. The seagrass at Gaya Island in Sabah declined because of heavy loads of suspended sediments, which reduced the sub-surface light intensity. Human activities involve dynamite fishing and construction, which are directly responsible for suspended sediments and water turbidity in coastal waters and seagrass habitat decline [19]. Furthermore, the seagrass at Pengkalan Nangka in Kelantan, Paka in Terengganu, and Punang-Sari Lawas in Sarawak degraded in term of occurrences because of human activities such as sand mining, heavy loads of suspended sediments, heavy rains, and floods. Due to this reason, efforts to systematically manage and map seagrass habitat in Malaysia is needed to map and monitor the seagrass ecosystems.

In the last few decades, scientists have been using underwater acoustic survey technologies to establish the relationship between geomorphological characteristics and benthic communities [20–25]. Multibeam echosounders (MBES), which are able to perform full-coverage mapping with a high spatial resolution dataset, have been used for the development of marine habitat suitability maps for fishes [26, 27], corals [28–32], starfishes, and crinoids [31], seagrass [33], and kelp [34]. Bathymetry maps and backscatter mosaics are the two primary products from MBES that can be used as surrogates for mapping patterns and processes on the seafloor (e.g., seafloor morphology and sediment composition) that influence the distribution of marine habitats. Bathymetry provides information on seafloor depth and multiple measures of seafloor complexity through terrain analysis [35]. The applications of bathymetry data, together with a wide range of physical predictors (e.g., aspect, slope, and rugosity) in mapping habitat distribution, have been demonstrated in previous studies [26, 31, 35–38], revealing the relationship between the acoustic measurements of seabed types and benthic habitats [39–41]. Backscatter data can provide information on different substrates by referring to different intensity levels [42]. These data have been used to identify the substratum type of seafloor in many habitat suitability predictions [31, 43].

Various modeling methods using bathymetry, backscatter, and their secondary derivatives (constructed from bathymetry or backscatter) for habitat suitability model (HSM) have been used previously [44–46]. These techniques have differed widely, in terms of the implemented algorithms and input data features. Combining these techniques with in-situ data to characterize the physical and biological datasets makes it possible to produce accurate HSMs [47]. Habitat suitability modeling is a widely used technique for predicting the spatial distribution of species and has been implemented in benthic-related studies [44, 48–50]. Habitat suitability modeling generally quantifies the relationship between occurrence data and predictors to explore the spatial distribution of a species and the response curve in relation to environmental factors [51–54].

The selection of a suitable spatial resolution dataset is important in marine environments [55–57]. High spatial resolution data will produce maps with detailed information, which is useful for detailed marine spatial management and planning [55]. In contrast, a low spatial resolution dataset might be used in a biogeographic study that involved measurement and monitoring patterns of species richness across broad spatial extents [58], such as influencing the accuracy of the HSM and management effectiveness in the marine environment [59]. Previous studies [50, 60] have conducted HSMs using several spatial resolutions by utilizing predictors extracted from various types of data sources. Spatial resolution was found to influence the correlation among predictors, model performance, suitability of predictors to predict habitat, and the spatial pattern of habitat suitability. Investigations of a suitable spatial resolution in habitat suitability studies are still limited; however, choosing the appropriate spatial resolution is expected to result in more meaningful habitat models [61].

Predicted habitats may also theoretically react to predictors for different analysis window sizes [27, 62]. However, there is limited understanding of the optimal window size for interpreting the relationship between species and habitat [63]. Associated predictors (e.g., bathymetric and backscatter predictors) are normally computed for specific analysis window sizes [44, 64]. Predictors are extracted from the primary data (e.g., bathymetry map and backscatter mosaic) using focal or neighborhood cell analysis. The derivation of predictors from the primary data at particular sizes of analysis windows (e.g., 3 × 3 or 9 × 9 cells) may not be sufficient to reflect the processes of interest [65, 66]. Furthermore, to our knowledge, no specific study has examined the effect of these window sizes on seagrass HSM. Thus, a detailed study is needed to investigate the proper analysis window size for seagrass HSMs. Adopting a multiscale approach to mapping seagrass habitats should ensure the capture of relevant scale-dependent predictors of ecological patterns and processes [67].

The primary objective of this study was to demonstrate the effect of different spatial resolutions and analysis window sizes on the production of seagrass habitat suitability maps. First, we collected and processed the bathymetry and backscatter datasets from the MBES. Second, we constructed the secondary predictors from bathymetry and backscatter using several analysis window sizes (3 × 3, 9 × 9, and 21 × 21 cells) and different spatial resolutions (1 and 50 m). Finally, we compared the performance of various habitat suitability models built using maximum entropy (MaxEnt) and identified the contributions of each variable to the prediction of seagrass habitat.

## Materials and methods

### Study area

**Fig 1:**
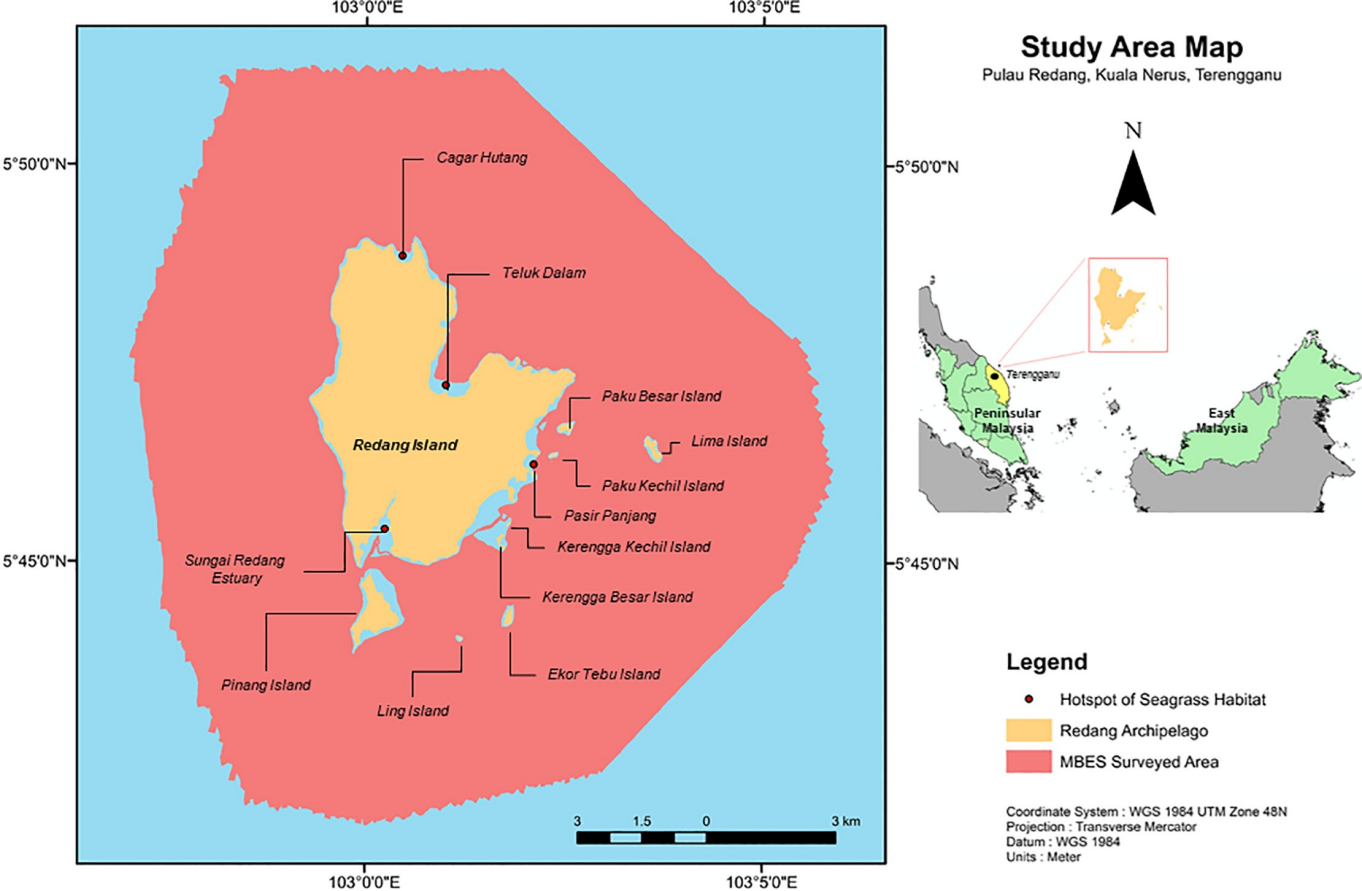
Location of the study area. Study areas are marked with a red dashed polygon which extends two nautical miles from the shoreline of Pulau Redang and other small islands.

The Redang archipelago is located in the South China Sea off the east coast of Peninsular Malaysia, in the state of Terengganu. It is located approximately 24 nautical miles off Terengganu’s coastline (Fig 1). Since 1993, the Department of Marine Park (now the Marine Park and Resource Management Division, Department of Fisheries Malaysia, Ministry of Agriculture and Food Industry) was responsible for protecting the offshore islands and their surrounding coastal waters. In the same, year under the Fisheries (Prohibited Areas) Regulations 1983 (Fisheries Act, 1963), the Redang archipelago was initially designated as a Fisheries Prohibited Area. With the establishment of the Marine Park Malaysia Order 1994 (Fisheries Act in 1995), the Redang archipelago was designated as a marine park, known as Redang Marine Park (RMP). The boundary of the RMP extends from the coastline (i.e., lowest sea level) up to two nautical miles and was established by the Marine Park and Resource Management Division [68]. This archipelago comprises Redang as the main island, together with eight small islands. The smaller islands are Pinang, Lima, Ekor Tebu, Kerengga Kecil, Kerengga Besar, Paku Besar, Paku Kecil, and Ling. Among these islands, Redang, Pinang, Lima, and Ekor Tebu have been established as part of the RMP.

The objective of marine parks is to provide protection to marine resources and habitats. Simultaneously, the marine park serves as a management tool to encourage sustainability in the marine ecosystem to ensure that marine resources are utilized sustainably [69]. Marine parks have been recognized to comprise at least one of these three main marine ecosystems: coral reefs, mangroves, or seagrass. RMP comprises two marine ecosystems: coral reefs [70] and seagrass [71]. Three seagrass species were recorded around the RMP from 13 species that were distributed over the broad areas of Peninsular Malaysia, including *Halophila decipiens*, *H*. *minor*, and *Halodule pinifolia* [71]. Most of these species can be found at Chagar Hutang, Redang River estuary, Pasir Panjang, and Teluk Dalam (Fig 1) and are generally distributed in subtidal areas. The species depth ranged from 2.5 to 24 m [71] and mainly occurred on the sedimentary substrate composed of silt and sand [71]. Equally important, Redang Island is known as a turtle nesting area [72, 73], and the H. decipiens meadows in the coastal area serve as feeding grounds for turtles [71].

### Data acquisition and processing

#### MBES survey

A Multibeam Echosounder (MBES) survey was carried out at two nautical miles from Redang Island and within the Redang archipelago (Fig 1) from April 6 to 24, 2019. The MBES dataset (e.g., bathymetry and backscatter) was acquired using a port -side mount Kongsberg EM2040C MBES system. Data logging, real-time quality assurance, display, and navigation were performed using Kongsberg Maritime’s acoustic data acquisition software, Seafloor Information System (SIS). The MBES operated at a frequency of 300 kHz, with different ping rates and pulse lengths that were automatically adjusted to the water depth in a high-density equidistant mode (400 beams per ping). Sound velocity profiles were collected using a sound velocity profiler once a day using a Valeport Monitor Sound Velocity Profiler. Tides were collected using a TideMaster Portable Tide Gauge at regular intervals of 10 min during MBES survey. The vessel’s position was acquired by a differential global positioning system (DGPS) mode using GPS/GLONASS corrections received by radio from the Fugro Marine Star satellite positioning service. A precise motion sensor system was used to measure the vessel motion data (e.g., heave, pitch, roll, yaw, and heading), which were recorded and set aside for data processing.

#### Ground-truth survey

Ground-truth data were recorded using a GoPro Hero 4 high-definition camera mounted on a stainless-steel frame. This frame was then dropped from the water surface to the seafloor, and the depth of each observation was ≤30 m. Within the MBES surveyed area, a cluster sampling technique was used in this study, where the targeted seagrass habitat locations were carefully chosen to capture all seagrass distributions based on a previous study [71]. The recorded video data were used to obtain video and photographic evidence of the occurrence of seagrass at each drop point. Surface positioning information was achieved using the AtlasLink H10 Smart Antenna. The position accuracy was improved by the DGPS method, which received the Atlas GNSS global correction service (accuracy of ± 50 cm). The expected error range for the true underwater position of each drop was estimated within a MBES pixel. Each recorded video data was analyzed and classified according to seagrass occurrence. All presence-only seagrass occurrence data were randomly selected for model training (75%) and model testing (25%).

#### Bathymetry data processing

**Fig 2:**
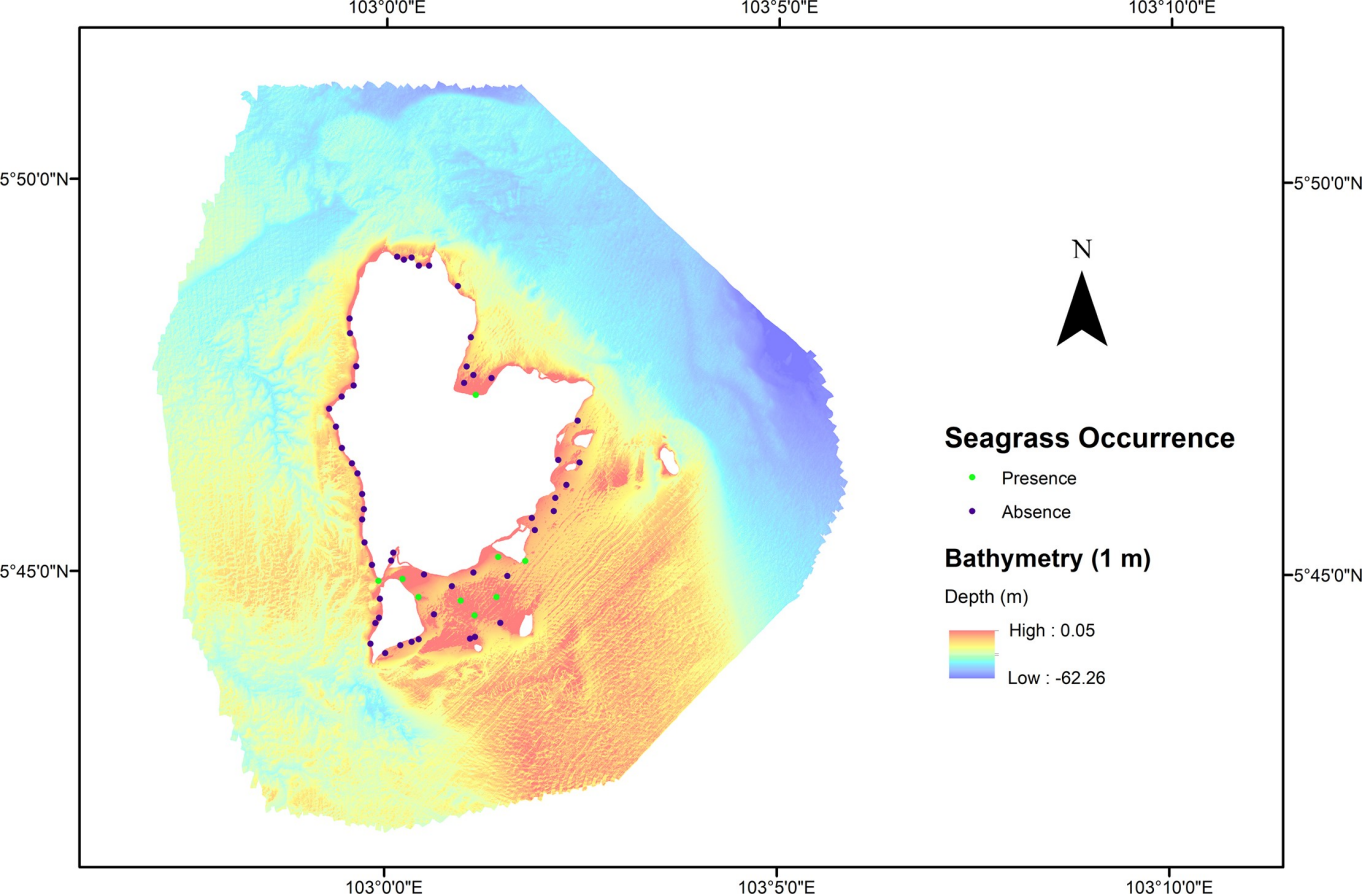
Bathymetry map of Redang archipelago. Bathymetry map of the study area. Red and blue dots represent the presence and absence of seagrass occurrence, respectively.

The raw MBES bathymetry data were processed using CARIS HIPS & SIPS version 10.4 following these steps: a) filtering of the position information using DGPS data, b) filtering of the motion information using attitude data (e.g., heading, heave, pitch, roll), c) depth correction using tidal observation data, and sound speed correction using sound velocity profiler data, d) data cleaning to remove uncertain data, and e) processed bathymetry data were gridded at several spatial resolutions, including 1 and 50 m (WGS 1984, UTM Zone 48 N) (Fig 2). The 1m dataset was used to test the effect of the high-resolution MBES dataset on the performance and prediction distribution of seagrass HSM. At the same time, 50m data was used to see the results of a low spatial resolution MBES dataset which is suited for regional studies (i.e., larger areas).

#### Backscatter data processing

**Fig 3:**
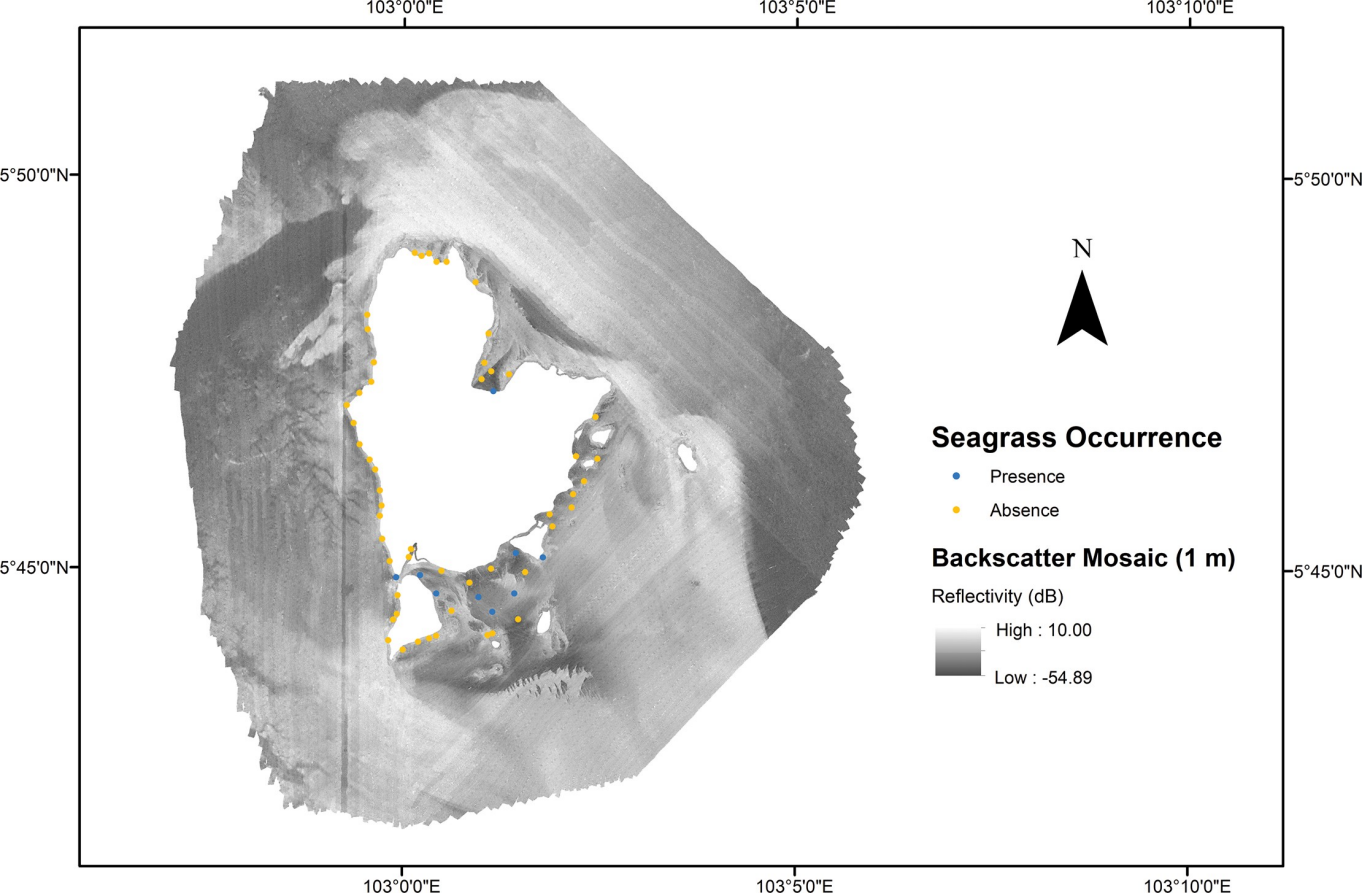
Backscatter mosaic of Redang archipelago. Backscatter mosaic of the study areas. Red and blue dots represent the presence and absence of seagrass occurrence, respectively.

The raw MBES backscatter data were processed using Fledermaus Geocoder Toolbox (FMGT) 7.4.4. The backscatter beam average data type was used as a backscatter data source, and all beams (400 beams) were used (starting and cut-off beam angles of 0° and 90°, respectively). The beam average was used to produce a backscatter mosaic image owing to some artifacts observed when processing the “beam time-series” data types. This was mostly due to the automatic mode used during MBES data acquisition (i.e., the pulse length is automatically adjusted according to the current water depth). The processing parameters remained closer to the FMGT default settings to maintain continuity between the surveys of the backscatter mosaics at the potential detriment of the subjective nature of each mosaic. Finally, the processed backscatter data were gridded to produce a backscatter mosaic at two spatial resolutions: 1 and 50 m (WGS1984, UTM Zone 48 N) (Fig 3). Two types of backscatter mosaics were used for this study: backscatter intensity presented in decibel (dB) values and backscatter intensity scaled to 8-bit grayscale values. The backscatter mosaic in dB values was the original intensity derived in FMGT while scaled intensity was used in the texture analysis. Apart from the mosaic, the prediction of sediment types and properties was also extracted using the angular range analysis (ARA) method in FMGT [74]. The mean grain size (phi) and sediment characterization were the two main outputs extracted from ARA processing and were used for this study. Each ARA parameter was exported to the raster format for further analysis.

#### Bathymetric predictors

A suite of bathymetric derivatives was obtained from the bathymetry maps to further describe the local variability within the MBES bathymetry data and delineate the topographic features [35, 59]. A set of four bathymetric predictors derived from bathymetry data were collected by the MBES survey using Geomorphometry for Ecology tool (Raster) in TASSE Toolbox v.1.1 [75]. The bathymetric predictors were derived using different surface analysis methods for extracting the geomorphological characteristics, such as slope, curvature [43], eastness, and northness [35, 38, 76, 77] (Table 1).

**Table 1:** Multiple analysis window sizes of bathymetric and backscatter predictors for seafloor morphology and sediment properties used in this study.

Predictors	Description	Analysis window size (cell)	Software
Bathymetry	Measures the depth (negative elevation) of the seafloor surface [79]	3 × 3, 9 × 9,	Focal Statistics (ArcGIS 10.5)
		21 × 21	
Slope	Rate of maximum change in depth from each cell of a surface raster in degree unit [80]	3 × 3, 9 × 9,	TASSE Toolbox v.1.1
		21 × 21	
Eastness	Measures the seafloor surface direction for the east and west directions	3 × 3, 9 × 9,	TASSE Toolbox v.1.1
		21 × 21	
Northness	Measures the seafloor surface direction for the north and south directions	3 × 3, 9 × 9,	TASSE Toolbox v.1.1
		21 × 21	
Curvature	Calculates the curvature of a raster surface [81]	3 × 3, 9 × 9,	TASSE Toolbox v.1.1
		21 × 21	
Backscatter Mosaic in decibels (dB)	Represents the acoustic intensity scattered by the seafloor [42]	3 × 3, 9 × 9,	Focal Statistics (ArcGIS 10.5)
		21 × 21	
Backscatter Mosaic 8-bit greyscale	Backscatter Mosaic was reduced into greyscale raster (0–255 units)	3 × 3, 9 × 9,	Focal Statistics (ArcGIS 10.5)
		21 × 21	
Homogeneity, Entropy, and Correlation	GLCM texture features predictors [78]	3 × 3, 9 × 9,	ENVI
		21 × 21	
Phi and Characterization	ARA-parameters for acoustic seafloor characterization [74]	-	FMGT

#### Backscatter predictors

Backscatter predictors (a continuous variable) characterize the acoustic reflectivity of the seafloor and are valuable surrogates for substratum features [27]. To further describe the local variability within the MBES backscatter data and delineate the seafloor substrate features, a suite of backscatter textural properties was produced from the backscatter mosaic (8-bit grayscale) using Haralick gray-level co-occurrence matrices (GLCMs) [24, 78]. The GLCM predictors were correlation, entropy, and homogeneity (Table 1).

#### Derivation of predictors at different analysis window sizes

The effect of analysis window size in bathymetric and backscatter predictors on the model predictions was tested by deriving all predictors at several analysis window sizes. This was completed using the focal statistics (mean) analysis function in ArcMap. Focal statistics (mean) analysis identified the average value of a variable within a specified window size centered on a given cell and positioned these data to the corresponding cell location. Three different window sizes (3 × 3, 9 × 9, and 21 × 21 cells) were used to mimic a range of analysis window sizes at which the topography (bathymetric predictors) and substratum (backscatter predictors) features might affect the distribution of seagrass (Table 1).

#### Correlation analysis of predictors

Correlations between the entire set of candidate predictors were initially reduced by excluding highly correlated predictors, based on the Pearson product-moment correlation coefficient [82]. The removal of high correlated predictors has been shown to be useful for habitat suitability model development in marine environment [31, 83]. Predictors with low correlations to other predictors which occurred in unique clusters were prioritized to avoid omitting information that was not already provided by other predictors and to avoid including only high-performing variables that provided similar information. Predictors with value correlation coefficients >0.5 were considered to have high correlation [84]. For each model prediction, individual predictor performance was tested using an iterative ’leave-one-out’ procedure (the best predictor was retained when the value correlation coefficients were <0.5). Predictors with value correlation coefficients of >0.5 were eliminated from the model development, and only low correlation predictors were used for further analysis.

#### Habitat suitability modeling

Habitat suitability modeling was carried out using maximum entropy (MaxEnt) species distribution modeling in MaxEnt software v3.4.1 [85]. MaxEnt used presence-only species occurrence data to estimate the probability of occurrence of a species that was used to distinguish between suitable and unsuitable areas [86]. MaxEnt has been found to be amongst the consistent methods with high accuracies for presence-only modelling in several marine studies [26, 87–95]. MaxEnt determined the probability distribution of the maximum entropy (i.e., the most dispersed or the closest to the uniform), and then constrained the distribution by utilizing a series of environmental predictors with a variety of values identified by the landscape at the locations where the species was known to occur [85]. MaxEnt is based on the premise that the unknown distribution of probabilities should have maximum entropy but is restricted by the niche’s environmental characteristics. MaxEnt controlled overfitting and variable selection using a regularization that smoothed out the distribution models, with a penalized maximum likelihood model that balances the model with the complexity of the model [49, 96]. MaxEnt was run using all seagrass occurrence data and selected MBES predictors. All models were using default settings in MaxEnt software, which were previously found to achieve excellent model performance [96–99]. For all models, auto features were used, which allows automatic limiting of feature types according to the amount of ground-truth data (i.e., seagrass presence data). The interaction between MBES predictors is automatically allowed to improve model performance. [96, 98, 99]. Twenty replicates and bootstrap procedures were used to obtain a firm model. Each replicate used randomly selected presence-only seagrass occurrence data for training data and test data (75% and 25% of the data, respectively) [98]. The output format portrayed in the logistic habitat suitability index ranged from the lowest "0" as low suitability for seagrass habitat to the highest "1" as high suitability for seagrass habitat.

In this study, the models that were trained with six different sets of predictors derived from MBES bathymetry and backscatter data were as follows:

- Model1_3- Uncorrelated predictors gridded at 1 m and derived using 3 × 3 analysis window size.
- Model1_9—Uncorrelated predictors gridded at 1 m and derived using 9 × 9 analysis window size.
- Model1_21—Uncorrelated predictors gridded at 1 m and derived using 21 × 21 of analysis window size.
- Model50_3—Uncorrelated predictors gridded at 50 m and derived using 3 × 3 analysis window size.
- Model50_9—Uncorrelated predictors gridded at 50 m and derived using 9 × 9 analysis window size.
- Model50_21—Uncorrelated predictors gridded at 50 m and derived using 21 × 21 of analysis window size.

#### Assessment of model performance

Receiver operating characteristic (ROC) curves were constructed, and the area under the curve (AUC) was used to compare the performance of the models [100]. The AUC is a test statistic that uses the presence and absence records to assess predictive model performance across the threshold ranges. This study utilized the Phillips approach [85] which applied randomly chosen pseudo-absences to ROC AUC rather than observed absences. The AUC was calculated based on the specificity and sensitivity of the predictive model. The specificity and sensitivity indicated the success rate for classifying suitable or less suitable seagrass habitats, respectively. The built-in function of the MaxEnt program produced mean AUC values of twenty replicates. AUC values >0.9 were indicated as excellent, 0.8–0.9 as very well, 0.7–0.8 as satisfactory and <0.7 represented poor discriminative ability [101, 102].

## Results

### Variable selection

This study found a weak correlation (<0.5) among several predictors derived from bathymetry data but not among those from backscatter data. The number and type of predictors with weak correlations varied between the models (Table 2). For Model1_3and 1_9, a weak correlation was found among bathymetry, slope, eastness, northness, and GLCM entropy with other predictors (S1 and S2 Figs). For Model1_21, only four predictors derived from bathymetry data such as slope, eastness, and northness were found to be uncorrelated predictors, among others (S3 Fig). For Model50_3, bathymetry, slope, and eastness derived from bathymetry data were found to be uncorrelated predictors, and GLCM correlation, GLCM entropy, and ARA characterization derived from backscatter data were found as uncorrelated predictors (S4 Fig). For Model50_9, low correlated predictors were found among bathymetry, slope, eastness, and northness derived from bathymetry data, and ARA characterization derived from backscatter data (S5 Fig). Bathymetry, slope, eastness, northness, and backscatter mosaic were observed as low correlation predictors in decibels (dB) for Model50_21 (S6 Fig).

**Table 2:** Low correlation between predictors for each model, Model1_3, Model1_9, Model1_21, Model50_3, Model50_9, and Model50_21extracted using Pearson Product-moment correlation coefficient.

Model1_3	Model1_9	Model1_21	Model50_3	Model50_9	Model50_21
Bathymetry	Bathymetry	Bathymetry	Bathymetry	Bathymetry	Bathymetry
Slope	Slope	Slope	Slope	Slope	Slope
Eastness	Eastness	Eastness	Eastness	Eastness	Eastness
Northness	Northness	Northness	*GLCM Correlation*	Northness	Northness
*GLCM Entropy*	*GLCM Correlation*		*GLCM Entropy ARA Characterization*	*ARA Characterization*	*Backscatter Mosaic in decibels (dB)*

^a^Italics indicate that the predictors were extracted from the backscatter data.

Predictors with <0.5 correlation variable (R^2)^ were considered weak and thus, included in the model.

### Seagrass presence from ground-truth

From the ground-truth dataset, 61 video drops were collected from the survey area. From all points, only nine points (red dots) were identified as having seagrass occurrences (Fig 2), while the remaining 52 drop points (blue dots) were absent data. Seagrass presence data were primarily found in the shallow water area with water depths ranging from 5.7 to 18.3 m (Fig 2). These areas displayed backscatter intensity levels between -14.1 and 22.0 dB (Fig 3).

### Model performance

The mean AUC values for the training dataset were 98% (1 m) and 96% (50 m), while the AUC values for the test dataset were 96% (1 m) and 94% (50 m) (Table 3). Model performance measured by mean training AUC and mean test AUC was generally excellent for training and test models. The AUC values indicated that training and test models showed a similar pattern of seagrass habitat suitability. In other words, the mean training and test AUC values of the high-resolution model were generally close to the results obtained by the low-resolution models.

**Table 3:** Training and test AUC values (mean) for both models and measured model performance for the high-resolution (1 m) and low-resolution model (50 m).

Model	Training Mean AUC (%)	Test Mean AUC (%)
Model1_3	98	93
Model1_9	98	93
Model1_21	98	97
Model50_3	99	95
Model50_9	99	98
Model50_21	99	98

Training and test AUC values (mean) described the model performance during model development and validation.

### Variable importance

**Fig 4:**
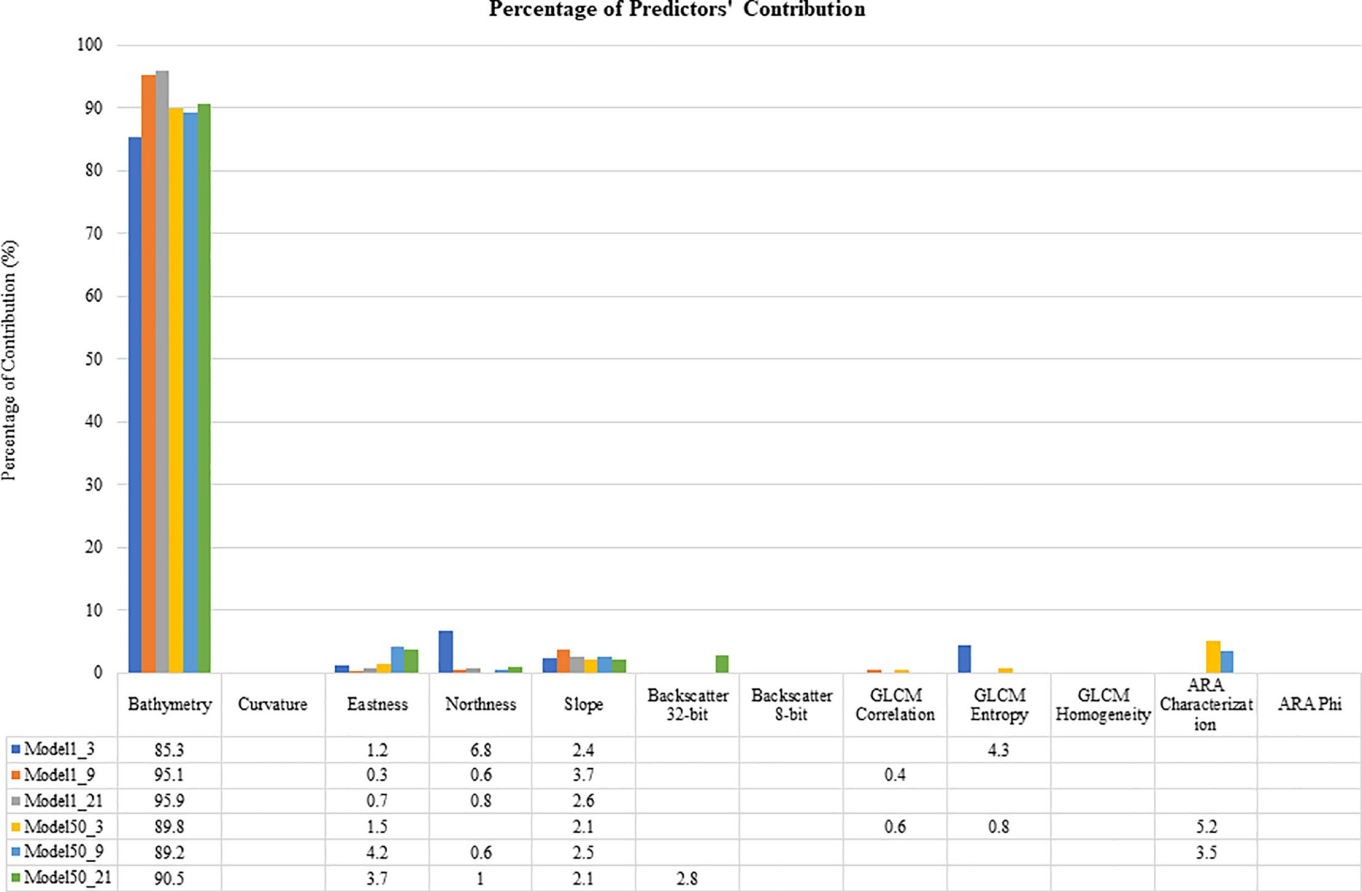
Relative contributions of the bathymetric and backscatter predictors by MaxEnt models. These predictors were used to build the seagrass habitat suitability models.

The study found that bathymetry was the most influential variable for all models, with the contribution value percentages for each model varying from 85.3% to 95.9% (Fig 4). The contributions of the other predictors were low and varied according to the model. Apart from bathymetry, the slope was listed in all the models. For backscatter predictors such as GLCM textures, GLCM entropy was the highest among the GLCM textures (4.3% in Model1_3). ARA characterization was the second-highest in Model50_3 and the third-highest contribution in Model50_9; however, it did not contribute to other models. Interestingly, the backscatter mosaic (8-bit grayscale) was only important in one model (Model50_21).

### Prediction models

**Fig 5:**
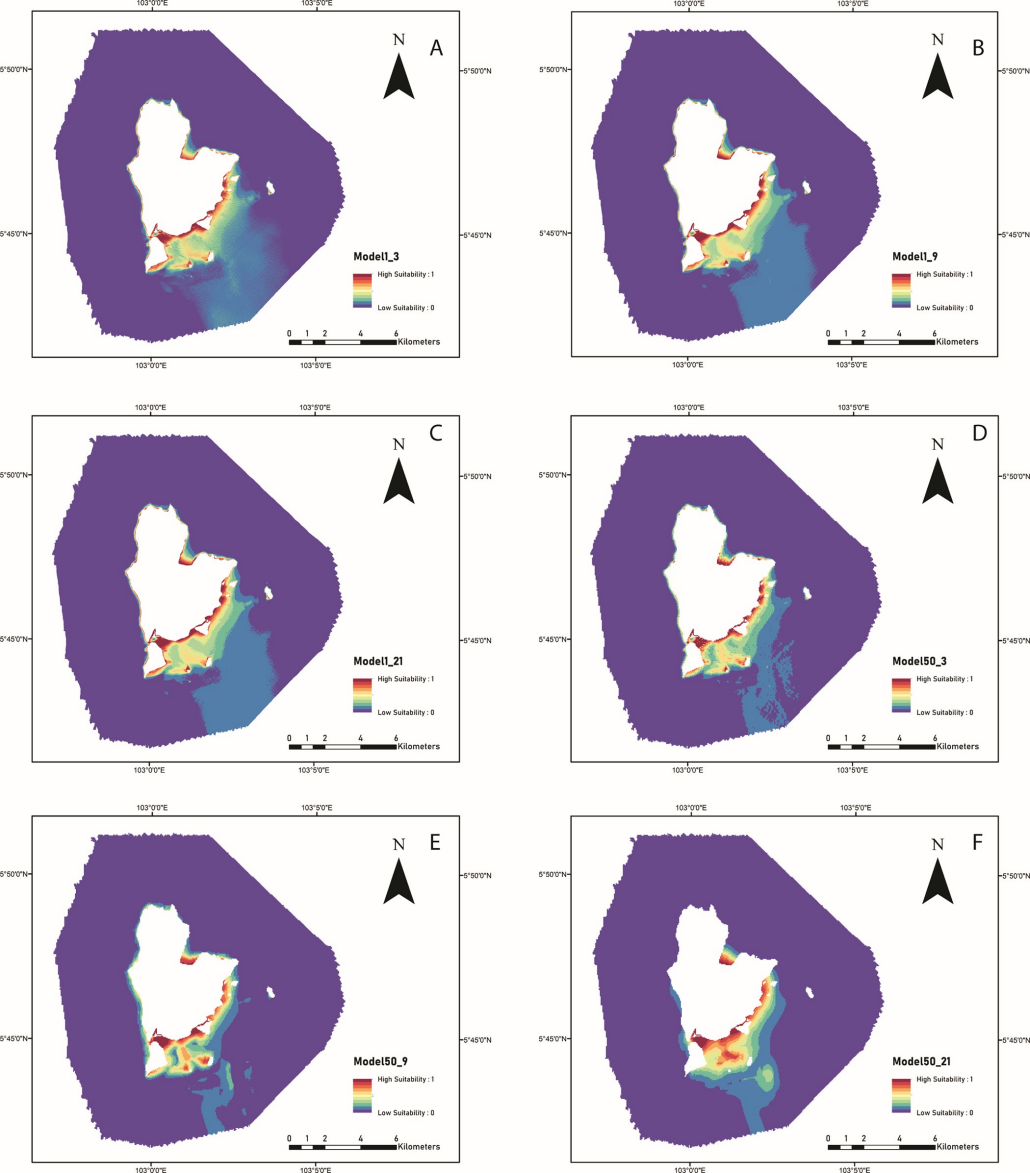
Model predictions on the suitability of the seagrass habitat across the MBES surveyed area. These models were produced using predictors derived from three analysis window sizes (n = 3, 9, and 21) and gridded at 1 m and 50 m; (A) 1 m (n = 3), (B) 1 m (n = 9), (C) 1 m (n = 21), (D) 50 m (n = 3), (E) 50 m (n = 9), and (F) 50 m (n = 21). The suitability index ranges from 1 (high suitability) to 0 (low suitability).

Generally, the prediction models (i.e., Models1_3, 1_9, 1_21, 50_3, and 50_9) gave similar results in predicting a suitable habitat for seagrass (Fig 5A–5E). Meanwhile, Model F showed significant spatial pattern differences in predicting the highest habitat suitability for seagrass (Fig 5F). Overall, the prediction models identified that seagrass habitats are broadly scattered across the shallow waters of the Redang archipelago. Areas of seagrass habitat were predicted to be in shallower (<20 m) waters and scattered between fringing reefs (east-south) (Fig 5). Some fragmented, seagrass habitats were also predicted throughout the shallower (<20 m) areas in the northwest of the prediction models and were scattered between fringing reefs (Fig 5). All models showed that seagrass was absent in deep water (> 20 m).

**Fig 6:**
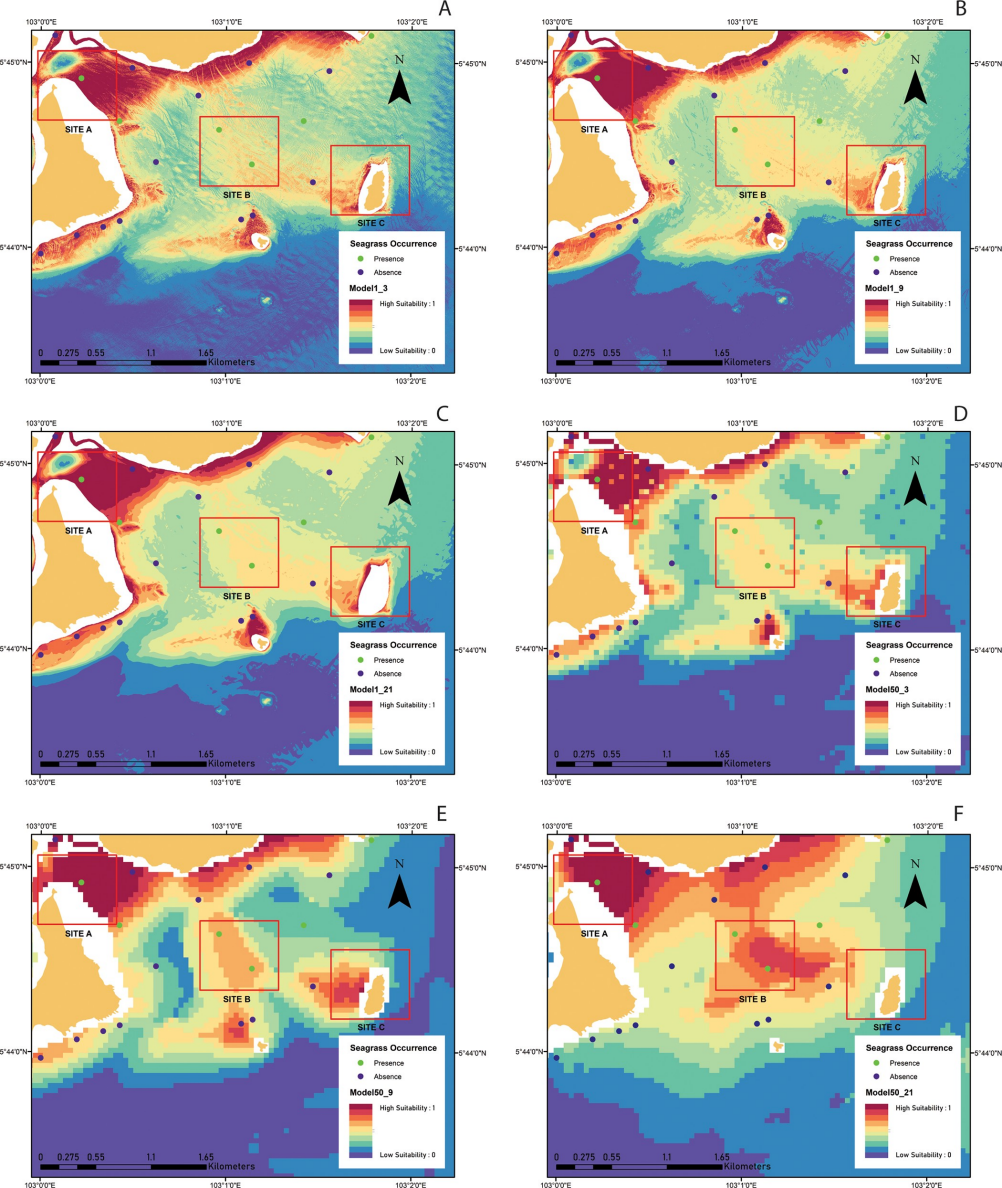
Model predictions of the suitability of seagrass habitat zoomed into three sites within the east-south area. Site A–northern area of Pulau Pinang; Site B–Cina Terjun cape; Site C–Pulau Ekor Tebu. The suitability index ranges from 1 (high suitability) to 0 (low suitability), while for spatial resolution and scale, (A) 1 m (n = 3), (B) 1 m (n = 9), (C) 1 m (n = 21), (D) 50 m (n = 3), (E) 50 m (n = 9), (F) 50 m (n = 21), respectively.

The effects of various spatial resolutions and analysis window sizes on the prediction models are illustrated in Fig 6. The models highlighted that the spatial pattern from all prediction models showed that the shallow area along the coastline primarily showed "high suitability for seagrass habitats ", except for the entrance to the Redang estuary (Site A). From the three small sites (sites A–northern area of Pinang Island, B–Cina Terjun cape, and C–Ekor Tebu Island), it can be seen that Models1_3, 1_9, and 1_21, showed a similar pattern of seagrass habitat suitability but were completely different from Models50_3, 50_9, and 50_21. This indicated that although all the prediction models were excellent (>90% accuracy), the habitat distribution did not necessarily agree with each other.

## Discussion

### Main findings

This study was among the first to explore the integration of high-resolution acoustic data, presence occurrence data, and the HSM to produce a seagrass habitat suitability map for RMP located within Malaysia’s coastal areas. Based on the produced HSMs, from 182.55 km^2^ of the total study area, 0.15–0.25 km^2^ were predicted as the most suitable habitat (suitability >0.8). Previous seagrass habitat investigations in the RMP area relied more on direct sampling methods, where no full coverage map was available [103]. In other marine parks in Malaysia, the probability estimation of seagrass occurrences has been produced through the extrapolation of the sparse point dataset at Tinggi Island [104]. The primary advantage of using acoustic data compared to conventional data (i.e., in-situ) is the ability of the former to obtain high-resolution spatial seafloor data, thus providing a more detailed representation of the habitat distributions. Although this technique was not entirely successful in revealing the seagrass at the species level (i.e., using the ground sampling technique), using acoustic data can produce a rapid and broad-scale seagrass habitat suitability map within the RMP for quick decision-making that does not require species-level accuracy. Habitat suitability map is also useful in directing further ground-truth surveys and pre-planning in other areas as well. Furthermore, this study focused on predicting the spatial distribution of seagrass habitat in the RMP as a function of the measured and modeled bathymetry and backscatter predictors of the area. This study demonstrated that the seagrass habitat was not randomly distributed, with depth being the most important variable for determining the most suitable habitats for seagrass. These results are supported by a previous study, which showed that the shallow subtidal zones in the inner bays (i.e., sheltered coastal waters) are dominated by fine sand [71]. However, this study appears to contradict with a previous study, where seagrass occurrence at Chagar Hutang occurred at <15 m in this study and 24 m in the previous study [71]. The presence of seagrass at different depths may be due to the vertical datum issue. The depth of seagrass suitability models was relatively reduced by vertical datum (i.e., Lowest Astronomical Tide). Meanwhile, in the previous study, the depth of seagrass locality points was relatively reduced by unknown vertical datum, which might be different from the present study. The results obtained in this study demonstrate that the seagrass habitat suitability maps are valuable in predicting seagrass habitats across the entire RMP.

### Predictor contribution

Among all predictors, bathymetry was the main contributor for all models. The results of this study were consistent with those of several previous studies that showed depth as an essential environmental parameter that limited seagrass habitats [71, 105, 106]. For example, seagrass habitats are known to colonize specific seafloor features in shallow water areas (< 24 m) and in the subtidal zone [11, 71]. Similarly, seagrass is a common species that inhabits shallow water over a range of depths and calm water that is sheltered from monsoonal effects [107]. Several studies have highlighted the importance of depth in the identification of seagrass habitat distributions [72, 106, 108]. Depth is a proxy variable for light attenuation [106] and temperature stress [109], and is directly significant to seagrass habitat distribution. Generally, the availability of sufficient light to support photosynthesis in seagrass depends on water depth [110]. Temperature also plays a critical role in controlling the growth rate of seagrass. Seagrass photosynthesis rates peak at an optimum temperature, and when it exceeds the optimum temperature, the photosynthesis efficiency declines rapidly [109, 111, 112]. Although no high-resolution temperature model from MBES is available, integrating the temperature derived from satellite imagery [113–117] for regional seagrass habitat suitability modeling might improve our understanding of the effect of climate change on seagrass distributions in the future.

Backscatter data, commonly used for the discrimination of seabed types and benthic communities, has been shown to improve HSMs [26]. While the use of bathymetric predictors to describe seabed characteristics is common in habitat suitability mapping using HSM, more proximal and functionally relevant predictors (e.g., backscatter predictors) are highly desirable for geological purposes. The present study revealed that adding backscatter predictors along with bathymetric predictors positively impacted model performance. The results from this study also supported that the analysis of the texture properties and angular backscatter intensity analysis (i.e., ARA) was more important than the backscatter mosaic itself. This was because most of the models used predictors from different analysis window sizes, which were almost similar to how GLCM predictors were constructed. Previous studies have shown that this signal-based method can differentiate between sediment classes using backscatter at different incidence angles. However, the contribution of these predictors was less important than that of bathymetry because the seagrass habitats are mainly occur where the sediment consists of fine sand that did not differ much in the study area (as predicted from the ARA technique). These results suggest that the contribution of backscatter predictors derived from different analysis methods could effectively capture the variation in sediment characteristics and should be integrated into the predictive modeling technique of marine species.

### Spatial resolution and analysis window size

Similar to terrestrial habitat mapping, identifying the ‘most suitable’ or ‘best’ analysis window sizes and resolutions to study marine habitat associations and habitat selection has not been performed [56]. Due to complex interactions between the physical characteristics of the seafloor, marine habitats have been considered difficult for predictive modeling [118] and to be mapped [119, 120]. This study showed that different analysis window sizes (e.g., 3, 9, and 21 cells) and resolutions (e.g., 1 and 50 m) had impacts on model performances and variable contributions. First, the contributions of most of the backscatter predictors could only be seen in the models with coarser spatial resolutions and less in the fine resolution models. One possible explanation is that the sediment type for seagrass with a higher suitability index was mostly sandy sediment that did not change significantly in these areas (as observed in Fig 6A–6C). Hence, the predictors from ARA (mean grain size and characterization types) were most likely similar and did not play an important role in predictive models A, B, and C (fine resolution), except for GLCM entropy and correlations. However, many of the contributions of backscatter predictors existed at coarser resolutions (Model50_3, 50_9, and 50_21), such as ARA characterization, GLCM (entropy and correlations), and backscatter intensity levels. This shows that the sediment type properties predicted from backscatter data were better observed at a coarser spatial resolution in this area. This suggests that backscatter data are useful for constructing predictive habitat models of marine species where complex sediment types are observed in a particular area. Second, although models with a coarser spatial resolution dataset achieved high accuracy, the spatial distribution pattern from these habitat suitability maps was not consistent (i.e., especially areas with high suitability indexes for sites A, B, and C in Fig 6). For Model50_3, it produced many small patches (Fig 6D) in which the habitat suitability values were not consistent with the adjacent areas; this problem was not observed in analysis window sizes of 9 × 9 (Fig 6E) and 21 × 21 (Fig 6F). However, the spatial distributions of seagrass suitability between these two analysis window size settings were completely different, as observed at sites B and C (Fig 6E and 6F). In other words, the effect of different analysis window sizes at coarser resolutions was more dominant than that of the fine resolution dataset. This highlights that the use of a high spatial resolution dataset is important for producing consistent predictive maps in the marine environment. In some studies, coarser analysis window sizes and spatial resolution models have been shown to underrepresent the area of suitable habitat because the finer scale habitat features that drive the species distributions were not captured by the coarser data [121, 122] or vice versa [56]. Thus, a modeling approach using different analysis window sizes [123, 124] and spatial resolutions may be considered an essential strategy in habitat suitability modeling [56, 125]. These findings can assist the future use of MBES techniques in similar environments using the specific analysis window size and for seagrass habitat suitability mapping.

### Limitations and recommendations

This study has several limitations. First, this study only used one-time sampling integrated with bathymetric and backscatter predictors to produce seagrass HSM. Due to the seagrass life cycle and threats to seagrass, interval or continuous sampling is needed to produce a temporal (i.e., dynamic) seagrass HSM [126–129]. It is important to examine the distribution of seagrass habitats with a different biology and ecology of seagrass species [128–131]. Thus, further study is needed to produce dynamic HSM models based on the temporal cycle of seagrass habitat. The current study only considered a specified analysis window size for each model. Previous studies found that different analysis window sizes may affect the distribution of benthic communities on the seafloor [31]. Furthermore, different multi-scale methods for calculating terrain information using an analysis distance approach are generally successful in identifying distinct seabed terrain features and making mapping results more transferable and interpretable [124]. These approaches may outperform single analysis window size method for modeling seagrass habitat suitability. While this study only focuses on correlation coefficient analysis between predictors, spatial correlation analysis [132–134] might improve the selection of the most important predictors. In addition, the present study area extends deeper than the maximum depth of seagrass presence observations in which depth has been identified (i.e., bathymetric map) as the dominant predictor. Thus, further study is needed to investigate the effect when the study area extends deeper than the maximum depth of seagrass presence observations. The current study only attempted to predict seagrass habitat at RMP, a coastal area of Redang, which is an offshore island with fringing corals. In this study area, the seagrass habitat is only found in subtidal areas, 2.5 to 24 m depth, and silt-sand area [71]. Further studies are also required to predict seagrass habitats that are distributed in different coastal zones (e.g., intertidal, sub-tidal, and lagoon) and substrate compositions (e.g., sandy-mud, fine sand, and muddy sand) [11, 71] along the coastal area of Peninsular Malaysia (e.g., west and east coast) and East Malaysia (e.g., Sabah and Sarawak). Furthermore, the current study only focused on acoustic data captured using a swath mapping system, while seagrass distributions are also dependent on other physical parameters [135] such as light availability [136], temperature [137, 138], and waves [3, 71, 139–141]. Combinations of these parameters will influence the prediction of spatial patterns of seagrass habitats in marine environments [139, 142]. In terms of spatial data coverage, MBES dataset gaps existed in shallow areas (<5 m depth) due to navigation safety and time restrictions considerations when using survey vessels as the MBES platform. Thus, satellite imagery, hyperspectral imagery, unmanned aerial vehicles, and airborne bathymetric lidar can be alternatives for producing seagrass HSMs that occur in these shallow areas [33, 143–145]. The current study only developed seagrass HSMs by using a maximum entropy modeling technique. Although this technique showed promising results [93, 94, 146], there are modeling techniques such as ecological-niche factor analysis [27], random forest, boosted regression tree, and generalized additive model [31] which are reported to perform well. Thus, future study is required to develop seagrass HSM by using several modeling techniques. Comparison of different modelling techniques with similar datasets can provide insight into different HSM performances or combine multiple methods into a single ‘best’ model, such as an ensemble forecasting approach to species distribution [147]. Current study only used the ROC AUC to assess the accuracy model where different validation measures can be used to assess the model performance, such as sensitivity and specificity, and the true skill statistic (TSS) for measuring the efficiency of species distribution models [100, 148].

## Conclusion

The present study has shown the applicability and usefulness of integrating an acoustic dataset from MBES, species occurrence data, and HSM techniques that could be used for seagrass habitat modeling at RMP. As high spatial resolution data were used, a detailed suitability map was produced where the information of the most suitable habitat for seagrass could be identified. With different spatial resolution data tested in this study, the results from the fine resolution dataset produced consistent suitability maps as compared to the coarser resolution dataset, although similar accuracies were observed. This study also concluded that the depth from bathymetry was the most influential predictor, regardless of the analysis window size and spatial resolution. In addition, a comparison of the spatial distribution of predicted seagrass habitat built using various spatial resolutions and analysis window sizes used in this study, may offer insight into the impacts of local-scale variation of seagrass habitat. Finally, the findings of the present study provide useful information for marine spatial planning for seagrass habitat and RMP management in Malaysian coastal waters using MBES data. This detailed seascape information is needed for designing field efforts to identify seagrass habitat localities and assessment of the distribution of seagrass habitat under protection and conservation management strategies at RMP.

## Supporting information

S1 Fig Pearson’s correlation coefficients between predictors on all presence occurrence data (n = 9) for Model A (spatial resolution = 1m & analysis window size = 3). Correlations ≥ 0.5 were emphasised.
(TIF)

S2 Fig Pearson’s correlation coefficients between predictors on all presence occurrence data (n = 9) for Model B (spatial resolution = 1m & analysis window size = 9). Correlations ≥ 0.5 were emphasised.
(TIF)

S3 Fig Pearson’s correlation coefficients between predictors on all presence occurrence data (n = 9) for Model C (spatial resolution = 1m & analysis window size = 21). Correlations ≥ 0.5 were emphasised.
(TIF)

S4 Fig Pearson’s correlation coefficients between predictors on all presence occurrence data (n = 9) for Model D (spatial resolution = 50m & analysis window size = 3). Correlations ≥ 0.5 were emphasised.
(TIF)

S5 Fig Pearson’s correlation coefficients between predictors on all presence occurrence data (n = 9) for Model E (spatial resolution = 50m & analysis window size = 9). Correlations ≥ 0.5 were emphasised.
(TIF)

S6 Fig Pearson’s correlation coefficients between predictors on all presence occurrence data (n = 9) for Model F (spatial resolution = 50m & analysis window size = 21). Correlations ≥ 0.5 were emphasised.
(TIF)

S7 Fig Receiver operating characteristic curves (ROC) was built and the area under the curve (AUC) for Model A. Mean training AUC value is 0.98 were indicated as excellent discriminative ability.
(TIFF)

S8 Fig Receiver operating characteristic curves (ROC) was built and the area under the curve (AUC) for Model B. Mean training AUC value is 0.98 were indicated as excellent discriminative ability.
(TIFF)

S9 Fig Receiver operating characteristic curves (ROC) was built and the area under the curve (AUC) for Model C. Mean training AUC value is 0.98 were indicated as excellent discriminative ability.
(TIFF)

S10 Fig Receiver operating characteristic curves (ROC) was built and the area under the curve (AUC) for Model D. Mean training AUC value is 0.99 were indicated as excellent discriminative ability.
(TIFF)

S11 Fig Receiver operating characteristic curves (ROC) was built and the area under the curve (AUC) for Model E. Mean training AUC value is 0.99 were indicated as excellent discriminative ability.
(TIFF)

S12 Fig Receiver operating characteristic curves (ROC) was built and the area under the curve (AUC) for Model F. Mean training AUC value is 0.99 were indicated as excellent discriminative ability.
(TIFF)
