# Supplementary figures and images for: Seagrass habitat suitability model for Redang Marine Park using multibeam echosounder data: Testing different spatial resolutions and analysis window sizes
Source: PLoS One. 2021 Sep 23;16(9):e0257761. doi: 10.1371/journal.pone.0257761 (PMC8459946; doi:10.1371/journal.pone.0257761)

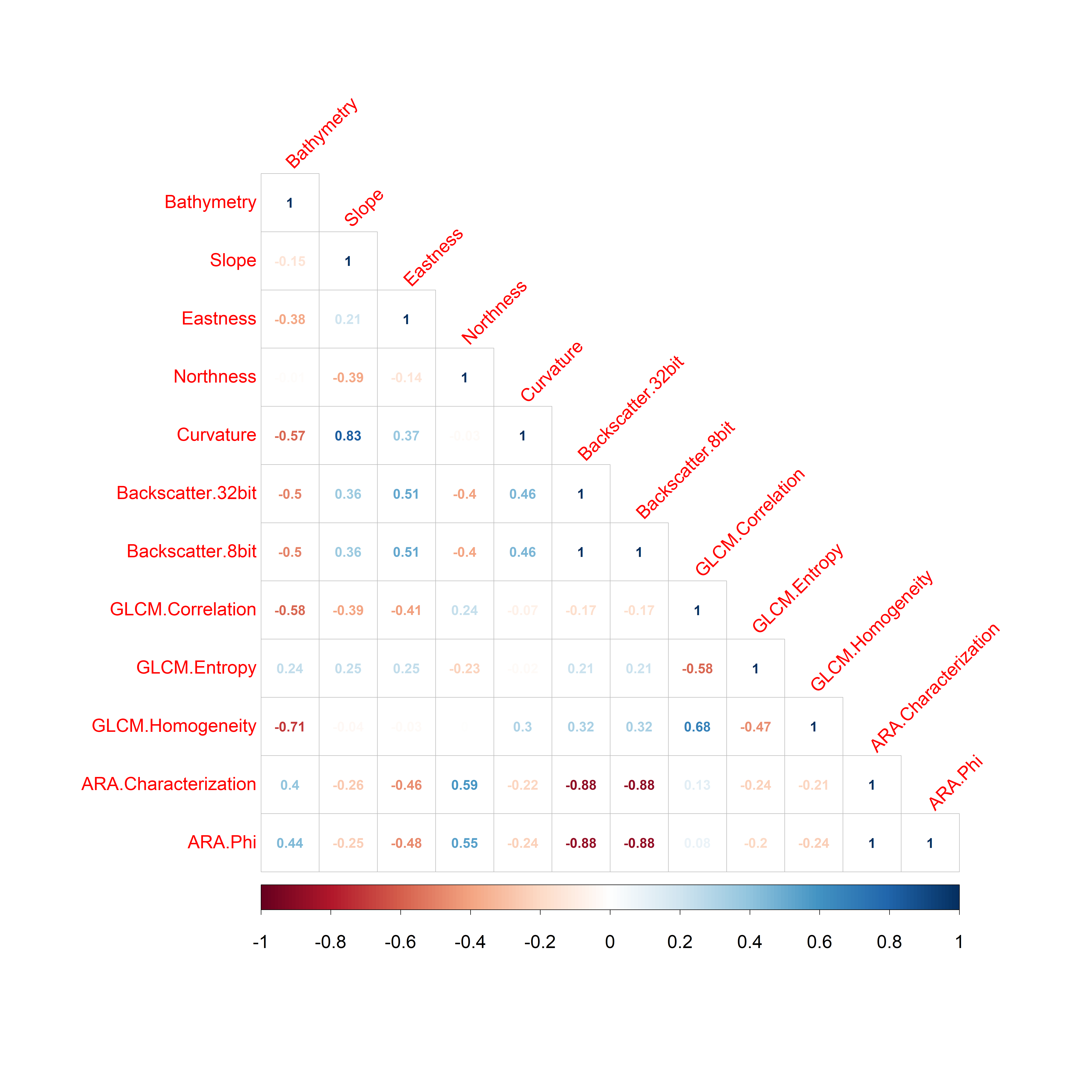

Supplement: S1 Fig — Correlations ≥ 0.5 were emphasised. (TIF) [file pone.0257761.s001.tif]

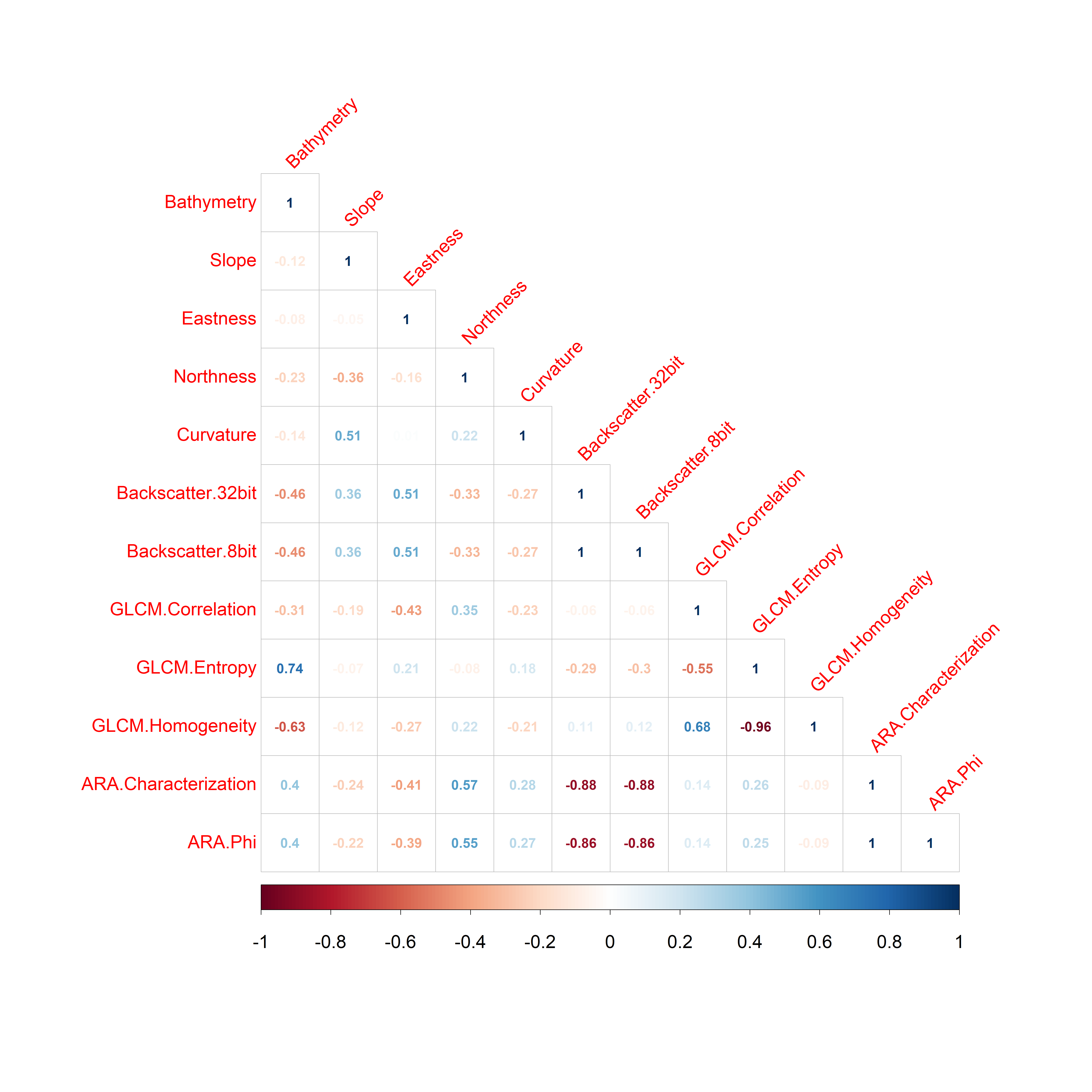

Supplement: S2 Fig — Correlations ≥ 0.5 were emphasised. (TIF) [file pone.0257761.s002.tif]

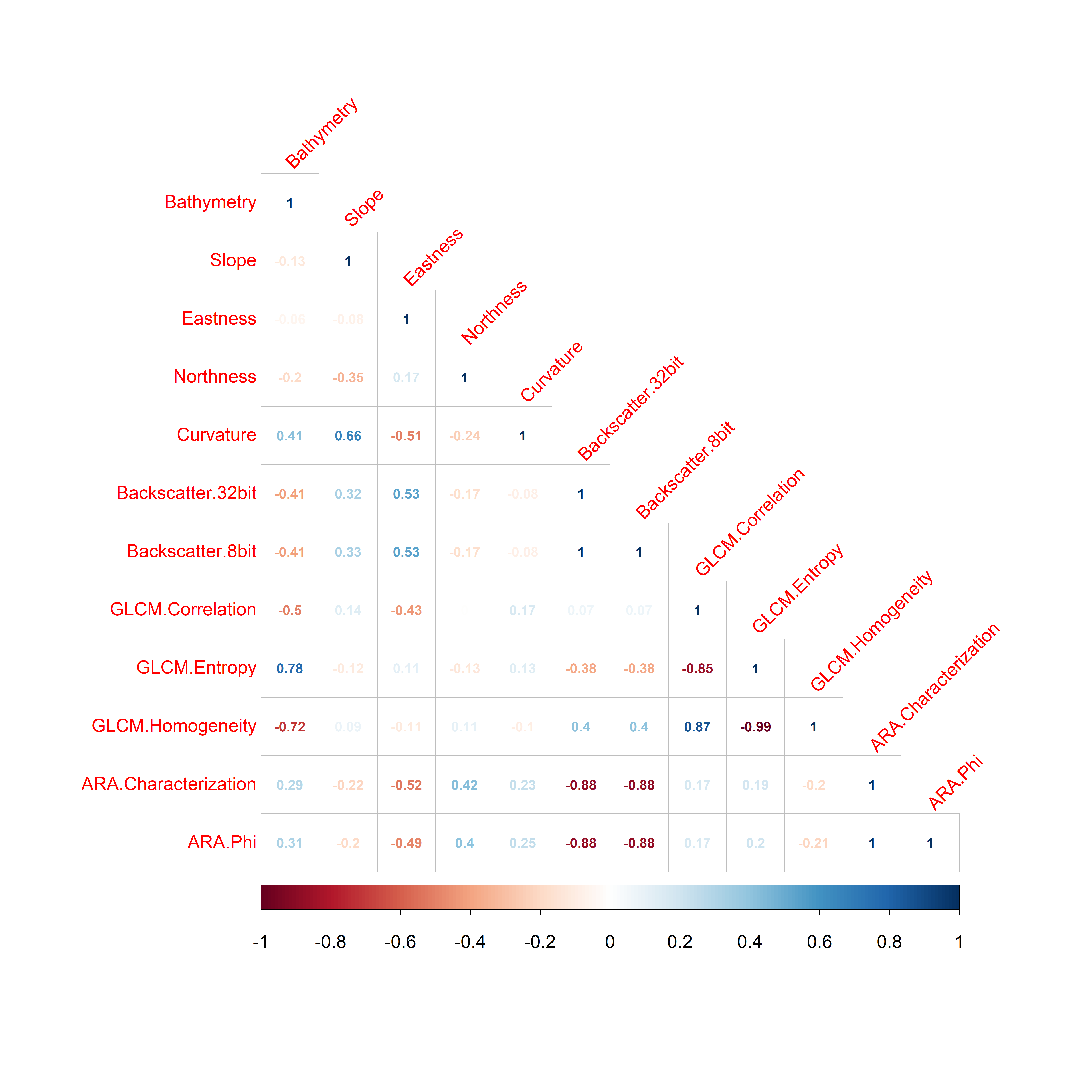

Supplement: S3 Fig — Correlations ≥ 0.5 were emphasised. (TIF) [file pone.0257761.s003.tif]

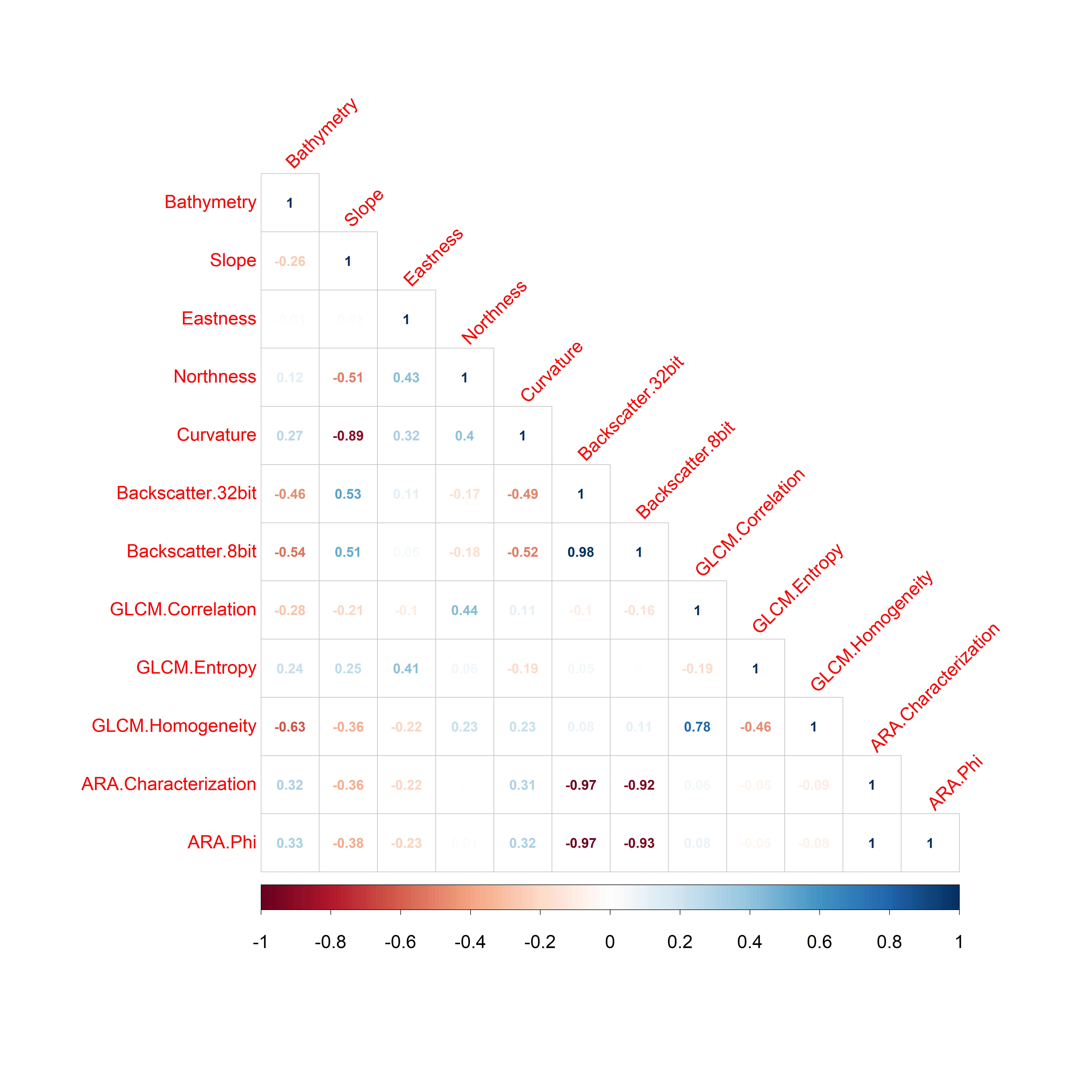

Supplement: S4 Fig — Correlations ≥ 0.5 were emphasised. (TIF) [file pone.0257761.s004.tif]

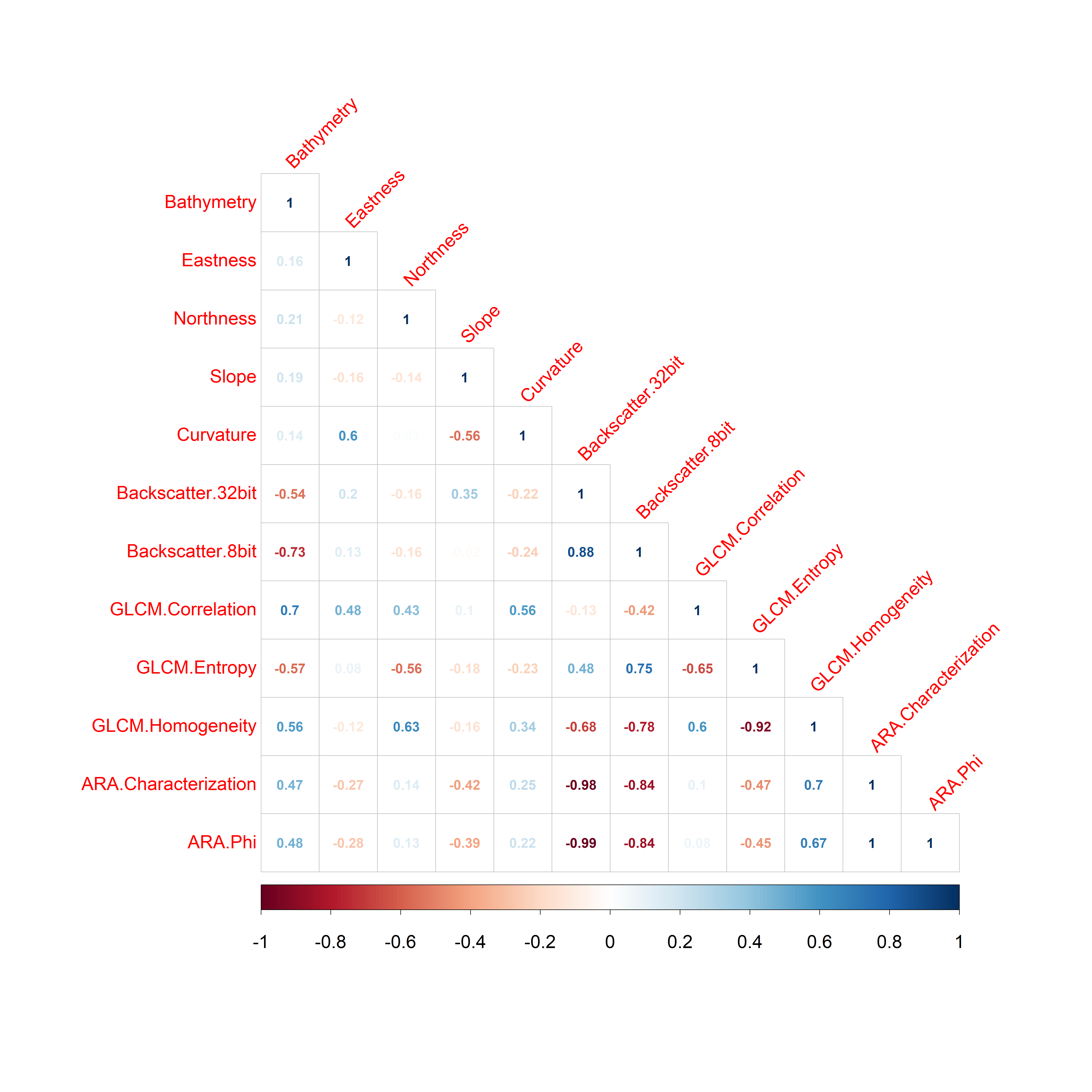

Supplement: S5 Fig — Correlations ≥ 0.5 were emphasised. (TIF) [file pone.0257761.s005.tif]

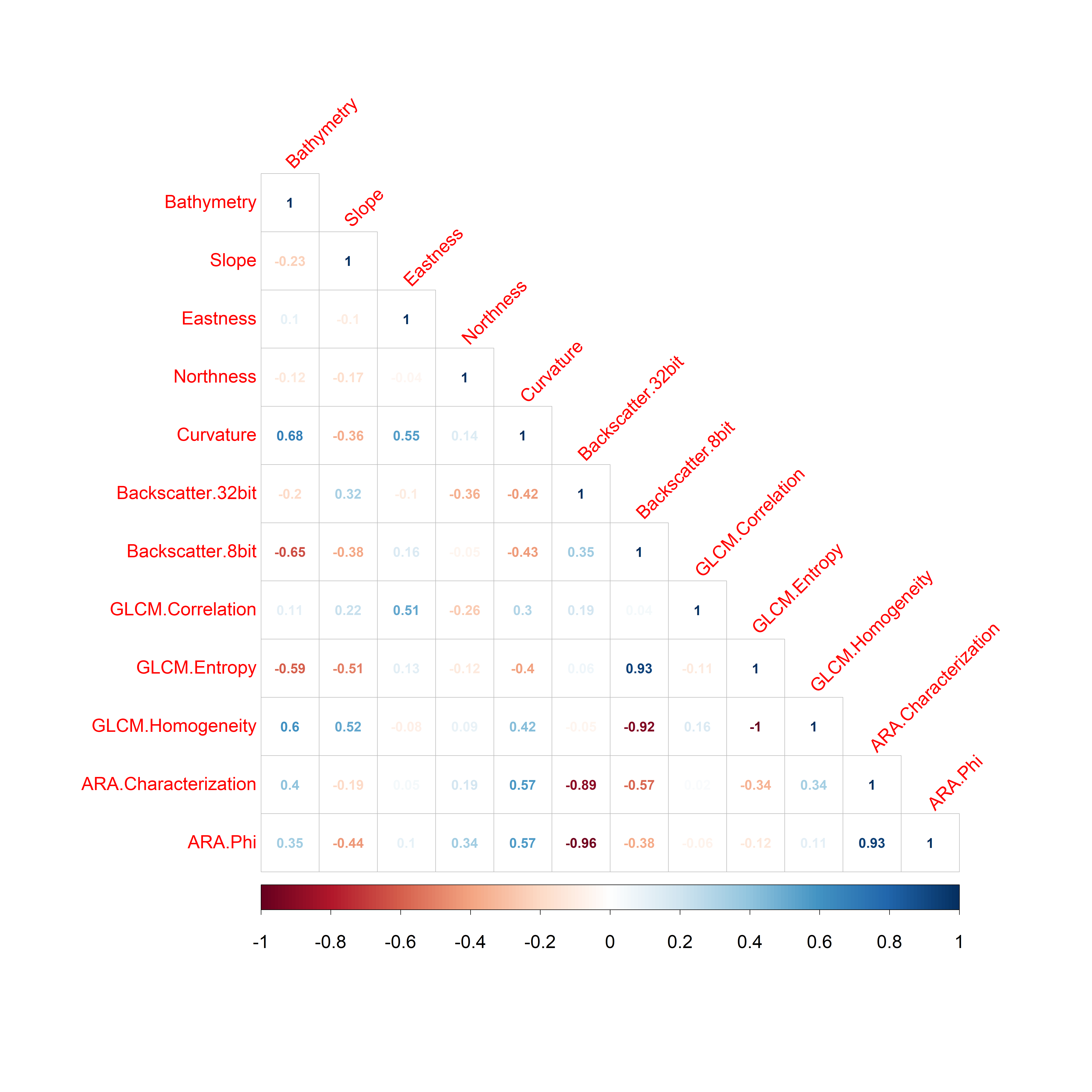

Supplement: S6 Fig — Correlations ≥ 0.5 were emphasised. (TIF) [file pone.0257761.s006.tif]

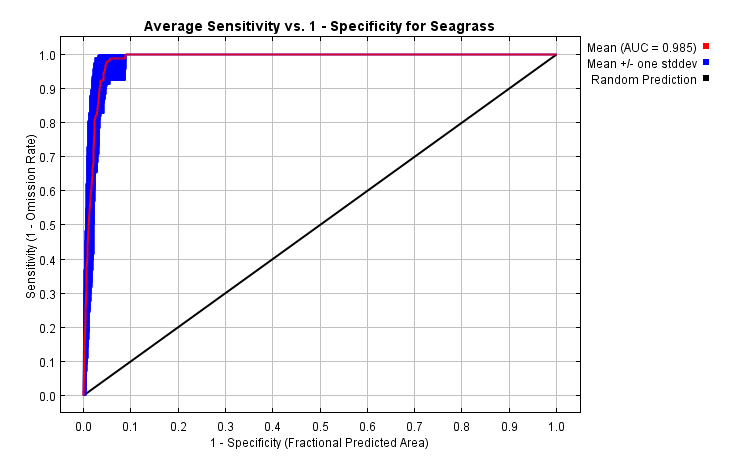

Supplement: S7 Fig — Mean training AUC value is 0.98 were indicated as excellent discriminative ability. (TIFF) [file pone.0257761.s007.tiff]

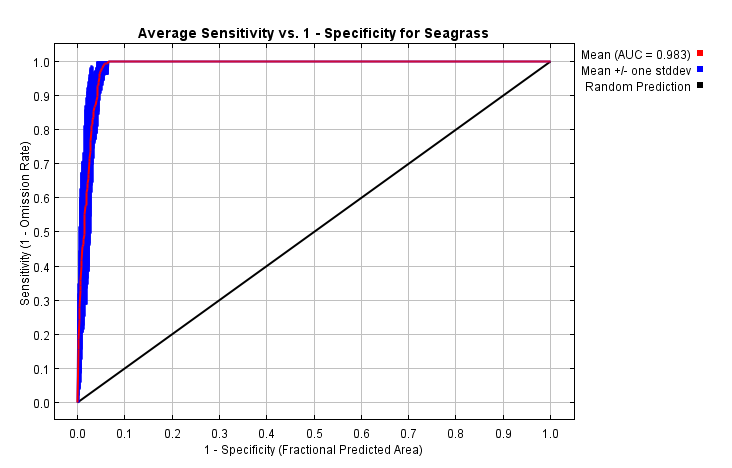

Supplement: S8 Fig — Mean training AUC value is 0.98 were indicated as excellent discriminative ability. (TIFF) [file pone.0257761.s008.tiff]

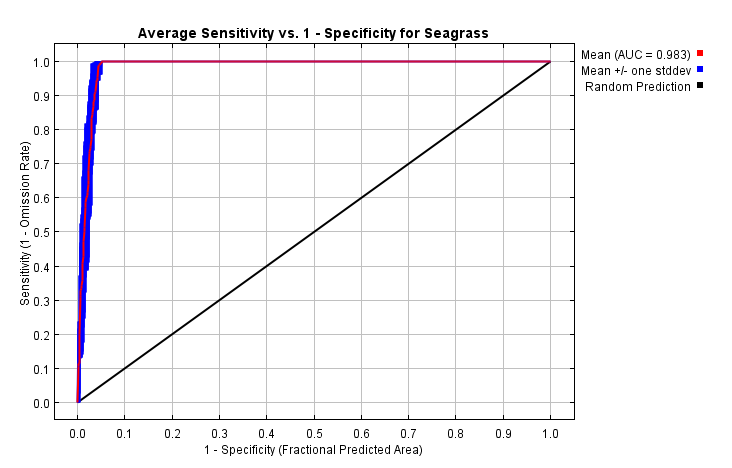

Supplement: S9 Fig — Mean training AUC value is 0.98 were indicated as excellent discriminative ability. (TIFF) [file pone.0257761.s009.tiff]

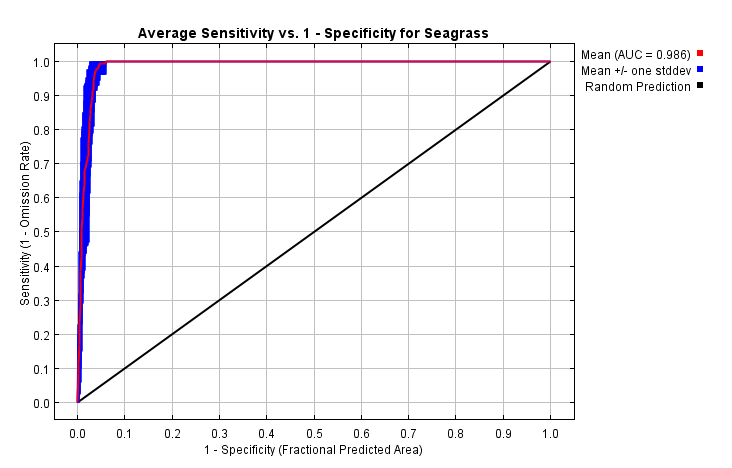

Supplement: S10 Fig — Mean training AUC value is 0.99 were indicated as excellent discriminative ability. (TIFF) [file pone.0257761.s010.tiff]

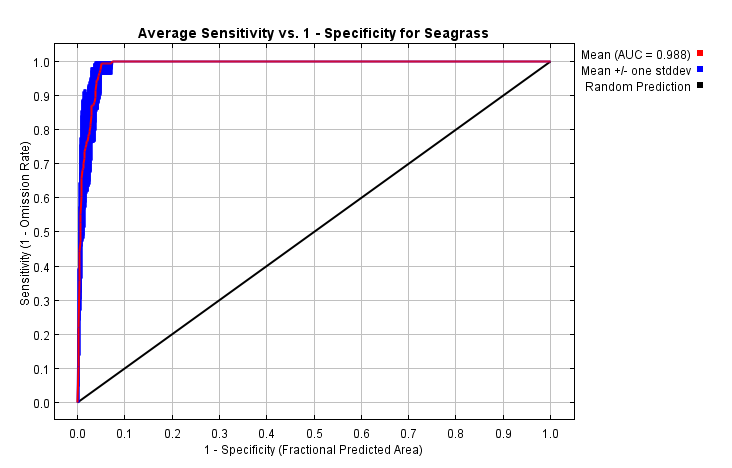

Supplement: S11 Fig — Mean training AUC value is 0.99 were indicated as excellent discriminative ability. (TIFF) [file pone.0257761.s011.tiff]

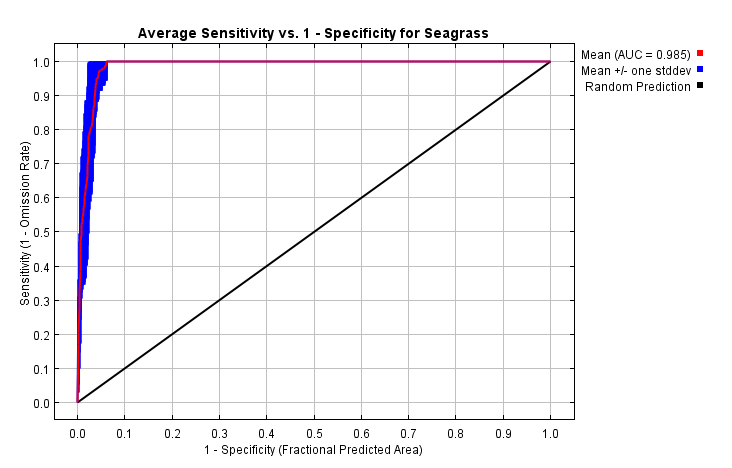

Supplement: S12 Fig — Mean training AUC value is 0.99 were indicated as excellent discriminative ability. (TIFF) [file pone.0257761.s012.tiff]
